# Complex Infection-Control Measures with Disinfectant Switch Help the Successful Early Control of Carbapenem-Resistant *Acinetobacter baumannii* Outbreak in Intensive Care Unit

**DOI:** 10.3390/antibiotics13090869

**Published:** 2024-09-11

**Authors:** Jozsef Kelemen, Marton Sztermen, Eva Krisztina Dakos, Jozsef Budai, Jozsef Katona, Zsuzsanna Szekeressy, Laszlo Sipos, Zoltan Papp, Balazs Stercz, Zsuzsanna A. Dunai, Bela Kocsis, Janos Juhasz, Fruzsina Michelisz, Zsuzsanna Daku, Judit Domokos, Dora Szabo, Lorand Eross

**Affiliations:** 1 Department of Neurosurgery and Neurointervention, Semmelweis University, 1083 Budapest, Hungary; 2 Gyula Nyírő National Institute of Psychiatry and Addiction, 1135 Budapest, Hungary; 3 Institute of Medical Microbiology, Semmelweis University, 1089 Budapest, Hungary; 4 HUN-REN-SU Human Microbiota Research Group, 1052 Budapest, Hungary; 5 Faculty of Information Technology and Bionics, Péter Pázmány Catholic University, 1083 Budapest, Hungary

**Keywords:** carbapenem-resistant *Acinetobacter baumannii*, healthcare-associated infection, HAI, antimicrobial resistance, disinfectant resistance, infection control, infection prevention, multidrug-resistant organism, outbreak, intensive care unit

## Abstract

A carbapenem-resistant *Acinetobacter baumannii* (CRAB) outbreak in an intensive care unit (ICU) was contained by an improved infection-control measure that included a disinfectant policy. In our retrospective cohort study, we describe the epidemiological investigations and infection-control measures during this outbreak. Descriptive analysis was used to summarize patient demographics, neurological diseases, surgical treatment, underlying diseases, infection, and outcomes. In December 2023, two CARB-positive patients were observed in the ICU, and four more patients became CRAB-positive in January. During this outbreak, there was an overlap of hospitalization periods among the CRAB-positive patients, and CRAB was isolated from the environment; the isolated CRAB strain was identical. Infection-control measures, including hand hygiene, contact precautions and isolation, surveillance, decolonization, environmental cleaning, and disinfection, were reviewed and modified. The aim of this study was to examine the molecular background of the effectiveness of the disinfectant shift used during successful outbreak control. Experiments were carried out to study the phenotypic sensitivity and genetic background of different disinfectant agents. A thorough analysis of the detected CRAB strain included whole-genome sequencing (WGS), investigation of the *qacE* and *qacEΔ1* genes’ relative expression by qPCR after exposure to different disinfectant solutions, as well as an analysis of biofilm formation. WGS analysis of the CRAB strain identified that an ST2 high-risk clone was responsible for the outbreak, which produced OXA-83 and ADC-30 beta-lactamases; in addition, *qacE* and *qacEΔ1* genes were also detected, which confer resistance to disinfectants containing quaternary ammonium compounds (QACs). A qPCR analysis demonstrated that after exposure to different disinfectants, the gene expression levels of *qacE* and *qacEΔ1* increased and correlated with concentrations of QACs of disinfectants. During the outbreak, the standard-of-care QAC-based disinfectant was changed to a mainly alcohol-based agent in the ICU, which contributed to the successful control of this outbreak, and no additional patients were identified with CRAB. We conclude that continuous surveillance and hand hygiene training combined with fast identification and reaction to new cases, as well as an in-depth analysis of multidrug-resistant outbreak strains and investigation of their disinfectant tolerance/resistance during an outbreak, are essential to effectively control the spread of nosocomial pathogens. The smart policy of disinfectant agent selection played a crucial role in controlling the outbreak and ensuring patient safety in the ICU.

## 1. Introduction

Outbreaks induced by multidrug-resistant *Acinetobacter baumannii* have been reported in recent years with an increasing frequency, representing an alarming threat in hospital settings worldwide [1,2]. *A. baumannii* is a major nosocomial pathogen, as it causes various systemic and life-threatening infections (e.g., wound infections, bloodstream infections, urinary tract infections, ventilator-associated pneumonia, etc.) among hospitalized patients. *A. baumannii* belongs to the ESKAPE group of infections, which are difficult to treat and are associated with high mortality rates, as only a limited number of effective antibacterial agents are available to treat such infections [3,4,5,6,7,8,9,10]. The World Health Organization has published a priority list where carbapenem-resistant *A. baumannii* (CRAB) is in the critical group; therefore, research, development, and discovery of new antibacterial agents are needed in order to treat these infections [11].

The genome of *A. baumannii* has high variability, as it can acquire and accumulate different antibiotic-resistance genes; thus, multidrug-resistant strains can develop. Nowadays, the major concern is about CRAB, that additional high-risk *A. baumannii* clones could evolve as they exhibit multidrug resistance and have the ability to survive and persist over a longer term in the environment (e.g., hospital environments). The most frequently reported high-risk *A. baumannii* clones belong to the following sequence types (STs): ST1, ST2, and ST3. Having said that, the currently predominant high-risk clone worldwide is ST2 [12].

Carbapenem resistance in *A. baumannii* is mainly explained by the production of OXA-type beta-lactamases (e.g., OXA-23-like, OXA-24/40-like, OXA-51-like, and OXA-58-like), but metallo-beta-lactamases (e.g., NDM, IMP, and VIM) can also be acquired by horizontal gene transfer, and among them, the NDM type is the most frequently reported in *A. baumannii* [13,14,15]. Additionally, resistance to fluoroquinolones, aminoglycosides, and colistin has been reported worldwide with an increasing frequency [16,17,18]. Furthermore, resistance to disinfectants containing quaternary ammonium compounds (QACs) and antiseptics has also been scrutinized [19,20,21]. This is especially true since QACs are commonly used disinfectants that include a high number of different agents such as benzalkonium chloride, didecyl dimethylammonium chloride, and alkyl trimethyl ammonium chloride [21].

Biofilms are resilient structures that are widely found in various natural habitats. They are also present in healthcare settings, where they are associated with healthcare-associated infections (HAIs). Healthcare-setting-associated biofilms are generally less susceptible to antimicrobials and disinfectants that are primarily used to inhibit the initial formation than others applied in less challenging environments. Eliminating established biofilms often requires additional, more aggressive disinfectant strategies on top of regular cleaning agents [22]. Surface biofilms with multi-resistant bacteria are common sources of environmental pathogens and therefore are associated with HAIs. Additional factors also influence the effectiveness of disinfectants, cumulatively decreasing the efficiency of infection prevention and control [22].

Choosing the appropriate disinfectant is crucial for effective infection control. Disinfectants recommended for patient-care items include QACs, alcohol, glutaraldehyde, hydrogen peroxide, peracetic acid, sodium hypochlorite, iodophors, and phenolics. Further important parameters need to be specified to ensure effective and safe infection control and prevent HAIs at sufficient disinfectant concentrations and exposure times. In addition, understanding their mechanisms of action and proper administration are needed to reduce the infection risk of vulnerable patients.

It is important to highlight the importance of touchless, i.e., contact-free techniques or techniques with minimal direct contact in cleaning and disinfection. These are useful practices to reduce cross-contamination and improve hygiene and safety in healthcare facilities and other cleanliness-critical environments. Given the increased tolerance and resistance to disinfectants, non-contact disinfection techniques are coming to the forefront. Furthermore, the successful use of innovative techniques—for example, violet-blue LED lamps—is frequently reported [23,24].

The purpose of the current study was to determine the role of disinfectant resistance genes in the spread of a CRAB strain during the outbreak, given that CRAB outbreaks are usually prolonged, persisting for months or years in hospitals [25,26]. In this report, we present successful and rapid infection-control measures focusing on disinfectant resistance in the control of a CRAB outbreak in a neurosurgery intensive care unit and the subsequent strengthening of infection-control interventions. 

## 2. Results

### 2.1. Description of the Outbreak

**Figure 1:**
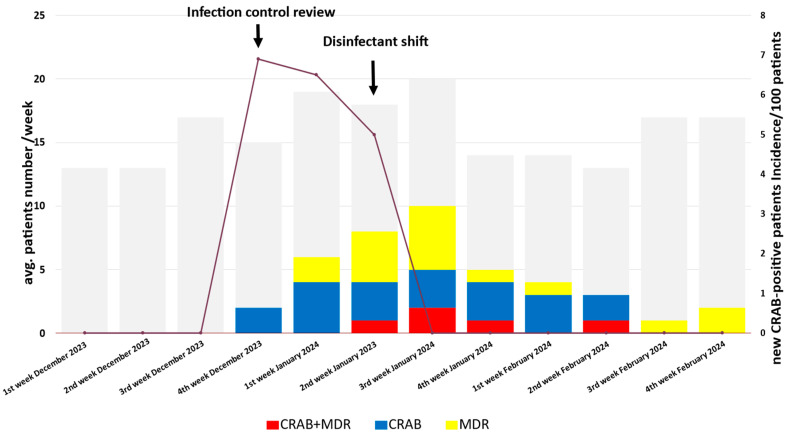
Timeline of the CRAB outbreak. The gray boxes indicate the number of patients treated each week in the ICU. The blue boxes represent patients with only CRAB.

Between 28 December 2023 and 22 February 2024, CRAB strains were isolated from six patients in the neurosurgical ICU at the Department of Neurosurgery and Neurointervention, Semmelweis University, Budapest, Hungary (Figure 1). The hospitalization periods of the CRAB-positive patients overlapped. The incidence of CRAB cases rapidly increased in December 2023 and moderately decreased in the first two weeks of January 2024. A rapid decrease in incidence was observed in the third week of January (Figure 1). Relevant background information on the patients treated in the neurosurgical ICU during this period is summarized in Table 1, and the characteristics of the six CRAB-positive patients are described in Table 2. Among the six patients infected during the outbreak, two patients were colonized, and four were infected with the newly appeared CRAB strain in the neurosurgical ICU. Five patients underwent craniotomy surgery, and one had a neurointerventional procedure. Clinical specimen sources of CRAB were trachea, blood, urine, liquor, wound, and anorectal swab. Of the six patients involved in this outbreak, three recovered from the CRAB infection; however, one patient died of a secondary bloodstream infection after a primary wound infection. Given that the patient with CRAB-positive wound infection was a contact person, we assumed that the CRAB wound infection was acquired in the ICU.

### 2.2. Environmental Investigations

There were no pathogen bacteria isolated from the surgery rooms and from any related surfaces, medical devices, or sinks. In contrast, at the ICU, different multidrug-resistant bacteria, including vancomycin-resistant Enterococcus (VRE) and extended-spectrum beta-lactamase (ESBL)-producing bacteria were detected on the different surfaces. VRE was isolated from the patient room surfaces, sink, sink drain, and excreta. The outbreak CRAB strain was detected in the excreta discharge, even after a few days of taking the samples. However, no CRAB was detected in follow-up tests conducted until June 2024.

### 2.3. Infection-Control Measures during the Outbreak

Upon the onset of this CRAB outbreak, existing infection-control interventions were reviewed, and a set of containment measures were instituted. Table 3 summarizes the infection-control measures used before, during, and after the CRAB outbreak.

During the CRAB outbreak, several measures were implemented to control the spread of the multidrug-resistant pathogen (Table 3). Hand hygiene education programs were held weekly, with daily monitoring of compliance. CRAB patients and positive contacts were isolated after diagnosis and occasionally housed in single rooms at the ICU. The patient:healthcare worker ratio was improved to 2:1 during the outbreak. Access by healthcare workers to the ICU was limited, and cohorting of patients and healthcare workers was implemented.

Throat decolonization with chlorhexidine digluconate was introduced. Environmental surveillance tests were conducted weekly in the ICU and surgery rooms.

Cleaning and disinfection procedures were enhanced with a checklist and increased frequency. The fourth-generation QAC disinfectant—didecyl dimethyl-ammonium chloride—has been constantly used in the surgery room. However, in the ICU, a first-generation QAC disinfectant—Alkyl (C12–16) dimethyl benzyl ammonium chloride (ADBAC [C12–18]), also known as MBF—was originally used. Patient zones were cleaned three times a day using both touch and touchless techniques.

In the ICU, a new disinfectant, named IP—predominantly containing 2-phenoxy-ethanol (10–20%) and benzalkonium chloride as active ingredients at 5–10% as a first-generation QAC component—replaced the previously used MBF disinfectant after a CRAB-positive patient and environmental sample were identified.

The introduction of the new disinfectant, IP, resulted in no new cases of CRAB. This intervention, together with environmental cleaning, disinfection, and surveillance, helped prevent the spread of CRAB during the outbreak.

### 2.4. Characteristics of CRAB Strains of This Outbreak

#### 2.4.1. Biofilm-Forming Capacity

According to the breakpoints observed using the microtiter-based method, the outbreak CRAB strains were found to be weak biofilm producer isolates.

#### 2.4.2. Antibiotic Susceptibility

Multidrug-resistant *A. baumannii* strains were isolated from six patients and from the environment during the outbreak. Antibiotic susceptibility testing revealed that all *A. baumannii* strains, both from clinical and environmental samples, exhibited identical resistance patterns. They were resistant to carbapenems, aminoglycosides, and fluoroquinolones but were susceptible to colistin with a 1 µg/mL minimum inhibitory concentration (MIC) value.

#### 2.4.3. Disinfectant Susceptibility of the Planktonic and Biofilm-Forming Phase of CRAB

The MIC and minimum bactericide concentration (MBC) were determined to be benzalkonium chlorate, 2-phenoxyethanol, and chlorhexidine digluconate for the planktonic phase of the outbreak CRAB strain. MIC values for both *A. baumannii* strains (the outbreak CRAB) and control *A. baumannii* BAA-1805 (purchased from ATCC and isolated from a Canadian soldier injured in Afghanistan) [27] were high, 16–32 µg/mL for benzalkonium chloride and chlorhexidine digluconate, and as expected, the MBC values were even higher, 32–64 µg/mL as well. However, the MIC values of 2-phenoxyethanol were significantly lower, 0.25 µg/mL for both the outbreak CRAB and for the control *A. baumannii* strains. For all investigated disinfectant agents, the MBC/MIC ratio was 1 or 2, indicating a bactericidal effect (Table 4).

Since the outbreak CRAB strain was found to be a weak biofilm producer, the minimum biofilm inhibitory concentration (MBIC) was determined. Benzalkonium chloride, 2-phenoxyethanol, and chlorhexidine digluconate inhibited biofilm formation of the outbreak CRAB strain at low concentrations. The phenomenon observed for MIC values was also observed for MBIC. The lowest MBIC value was measured for 2-phenoxy-ethanol, as 2phenoxy-ethanol inhibited biofilm formation to the greatest extent (Table 4).

#### 2.4.4. Killing Curve Assay with the Two Different Surface Disinfectants—MBF and IP—Used during the Outbreak

**Figure 2:**
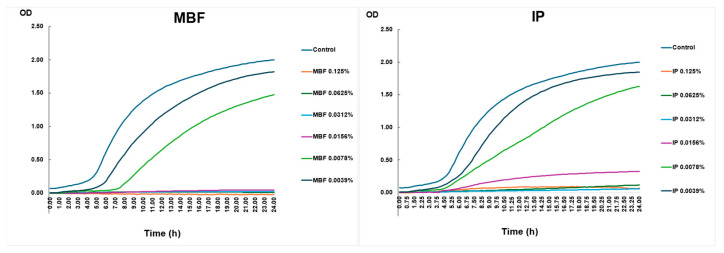
Killing curve assay.

An in vitro killing curve assay was performed to determine the growth capacity of the planktonic CRAB strain. The growth of the CRAB strain was investigated in the presence of two types of disinfectants. MBF, which contained a higher percentage (10–20%) of first-generation QAC, was used as surface disinfectant before the outbreak. During the outbreak, it was switched to IP, the active ingredient of which was 2-phenoxy-ethanol (10–20%), but it also contained 5–10% of first-generation QAC compounds. Upon exposure to sub-lethal concentrations of both MBF and IP surface disinfectant agents, the CRAB outbreak strain was able to grow at 0.039% and at 0.078% (Figure 2).

The growth curve of the planktonic CRAB strain is shown under different concentrations of surface disinfectants over 24 h. Bacterial growth was detected by measuring the OD. The figure’s left panel shows the rate of growth of different concentrations of MBF, and the right panel shows the increase in the values of IP.

#### 2.4.5. Genomic Analysis

**Figure 3:**
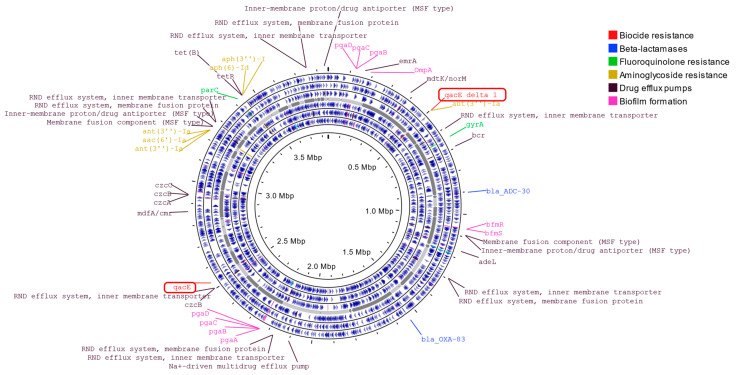
Whole-genome sequence analysis of *A. baumannii* strain of this outbreak.

In order to reveal the additional properties of the CRAB outbreak strain, whole-genome analysis was performed, and different antibiotic resistance genes were detected (Figure 3). OXA-83 and ADC-30 beta-lactamases, *aph(3″)-I*, *aph(3″)-Ia*, *aph(6)-Id,* and *aac(6″)-Ia* aminoglycoside resistance determinants, were identified. Furthermore, several efflux pump genes were observed to induce resistance against many antimicrobial agents. Additionally, *pgaA*, *pgaB*, *pgaC*, *bfmR*, *bfmS,* and *ompA* genes playing a role in biofilm formation were recognized (Table 5). Interestingly, acquired resistance genes, *qacE* and its variant *qacEΔ1*, were detected in the genome of CRAB. These genes code resistance to disinfectants that contain QACs.

#### 2.4.6. Real-Time PCR Assay to Determine the Relative Expression Rate of *qacE* and *qacEΔ1*

**Figure 4:**
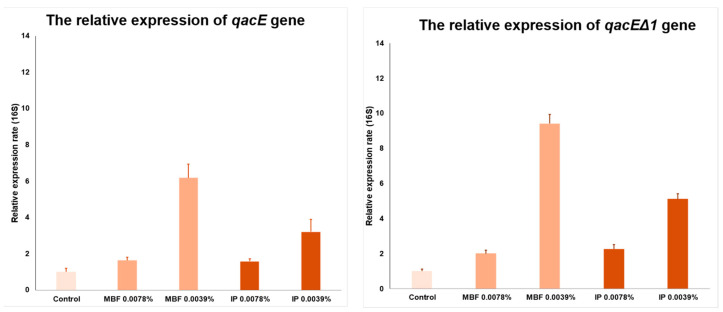
The real-time PCR assay.

Since at sub-lethal concentrations of 0.039% and 0.078% in the presence of both MBF and IP surface disinfectant agents, the CRAB outbreak strain was still able to grow, these two concentrations were used during the qPCR assay. In order to determine how the *qacE* and *qacEΔ1* genes allow the outbreak CRAB bacteria to adapt and develop tolerance to QACs at sub-lethal concentrations, real-time PCR was used to quantify the *qacE* and *qacEΔ1* relative expression rate. As a housekeeping gene, 16S was used. A sub-lethal 0.0078% concentration of both MBF and IP surface disinfectant agents hardly changed the relative expression rate of either *qacE* or *qacEΔ1*. However, at a sub-lethal 0.0039% concentration, MBF surface disinfectant agents significantly increased the relative expression of both *qacE* and *qacEΔ1*. The relative expression of the *qacE* gene increased six times, and that of the *qacEΔ1* gene increased >9 times (Figure 4). The simultaneous presence of the two genes, *qacE* and *qacEΔ1,* provides an additive effect.

The circos plot shows the GC content skew (inner circle with histograms). The SPAdes assembled contigs are plotted in gray (middle circle). The positions of all predicted genes are shown separately by reading frames (six circles with blue marks).

The relative expression of *qacE* (on the left) and *qacEΔ1* (on the right) genes in different concentrations, 0.0078% and 0.0039%, of the two surface disinfectants—MBF and IP—is shown.

Based on the relative expression, apparently, the expression of *qacE* and *qacEΔ1* genes are concentration-dependent and inducible. CRAB showed an increase at both 0.0078% and 0.0039% MBF and IP concentrations, but the expression of the *qacE* and *qacEΔ1* efflux pumps increased only at a 0.0039% concentration. The dependence of the relative expression on the concentration and active substance content is further confirmed by the fact that the difference between the increase in MBF and IP expression is proportional to the QAC content of the two disinfectants. MBF contains 10–20% and IP 5–10% QAC, and the *gacE* expression is increased by 6 times for MBF and by 3.2 times for IP. These values regarding *qacEΔ1* expression are increased 9.4-fold for MBF and 5.1-fold for IP.

## 3. Discussion

HAIs are commonplace but preventable events, affecting patients in healthcare settings worldwide. Enhancing the routine cleaning and disinfection procedures of hospital environments has been proven to lower the risk of HAIs [28]. The prevention and control of HAIs represent a major global public health challenge, raising concerns among healthcare workers, patients, and the public. This issue has become even more critical with the rise of multidrug-resistant pathogens.

The global incidence of antibiotic resistance in *A. baumannii* has increased steadily in the past 10 years, and it is associated with a higher burden of attributed mortality [29,30]. CRAB is a major concern in healthcare settings due to its high antibiotic resistance and persistence and due to its role in several outbreaks worldwide [30,31,32,33]. In order to control outbreaks caused by CRAB, several infection-control measures are applied. A multimodal infection prevention control approach appears to be superior, provided it is implemented as a ‘bundle’ of interventions [34,35,36].

The purpose of our present study was to determine the factors that led to the rapid and successful eradication of the CRAB outbreak that occurred at the ICU. We clarified the role of the disinfectant shift during outbreak control and investigated the presence and role of genes responsible for disinfectant resistance.

In our study, a multidrug-resistant *A. baumannii* ST2 high-risk clone induced an outbreak in a neurosurgery ICU, and during this outbreak, six patients were involved. We implemented different infection prevention control (IPC) measures, including hand hygiene, contact precautions, staff education, additional active screening, cohorting staff and patients, environmental cleaning and disinfection, monitoring of environmental cleaning, antimicrobial stewardship/monitoring of antibiotic consumption, active respiration, perianal screening, and environmental cultures. The effectiveness of the combination of these techniques has already been described by several studies [37,38,39,40,41,42,43]. Nasopharyngeal decolonization with chlorhexidine digluconate introduced during the outbreak in our ICU is another well-documented infection-control measure [44].

Contact precaution is generally applied during CRAB outbreaks; however, staff or nursing cohorting, as we implemented, has rarely been applied [43,45,46]. During IC measures, hand hygiene practices were implemented, and alcohol hand rubs were used; this practice, as a basic practice, is also commonly described in *A. baumannii* outbreaks [47]. Increased hand hygiene control and monitoring also contributed to our outbreak control, as the implementation of hand hygiene best practices was described by other studies as well [41,42,45].

CRAB isolates can survive in the hospital environment for a long time because of biofilm production, so they could be detected from colonized surfaces and environmental samples [47,48,49]. However, environmental sampling is not widely used and has only been performed in a few reports [43,46,49,50,51]. In our study, we could successfully culture bacteria from the sink, and we believe that its elimination could play a role in outbreak control.

During the outbreak, passive microbiological surveillance in the ICU was changed to active surveillance, with rectal screening, urine screening, and respiratory–pharynx, trachea–screening tests for all patients upon admission and every 5 days afterward. The high sensitivity of combined screening methods, rectal and pharyngeal screening [41], and rectal and skin screening is well documented [45].

Most infection-control activities are carried out during the control of outbreaks; however, very few studies detail cleaning and disinfection techniques [48]. Opinions differ on the effectiveness of touch and non-touch cleaning techniques. Controlling the efficiency of cleaning and disinfection is questionable and depends on the institution. In a recent review, it was concluded that the choice of disinfectant appears less critical than ensuring thorough cleaning and the complete removal of biofilm [52]. Our data, however, suggest that the disinfectant type used had a strong effect on the outcome of the outbreak. Due to the resistance to disinfectants, properly selected disinfectants have an important role in successful outbreak control. Because of the presence of *qacE* and *qacEΔ1* efflux pump genes and due to their inducibility, we recommend the use of disinfectant agents containing several different active ingredients as a main component to prevent the development of disinfectant resistance.

The correct choice of disinfectant policy, supplemented with additional infection-control measures, contributes significantly to the successful control of CRAB outbreaks.

We managed to control the CRAB outbreak after the introduction of a new, mainly alcohol-based disinfectant IP, which replaced the conventional QAC-based agent MBF. This measure resulted in the containment of the outbreak, and no further infection or dissemination was detected. These results pinpoint the importance of applying disinfectant agents with not only one but multiple active ingredients to render the emergence of resistance.

Unfortunately, it is not possible to routinely characterize the susceptibility to disinfectants of the outbreak strains in hospitals, and there are no breakpoints agreed upon based on international consensus for the standardized breakpoints for the determination of tolerance and resistance. In the future, biocide tolerance and resistance will be recognized sooner by fast detection either by PCR targeting to *qacE* and *qacEΔ1* or by whole-genome sequencing based on the available resources of the laboratory. As a result, appropriate infection-control measures, e.g., changing disinfectants, can take place as soon as possible and can play a significant role in controlling the outbreak.

A recent study analyzed antiseptic MIC values for *A. baumannii* clinical isolates and found that the presence of *qacE*, *qacEΔ1*, and *aceI* genes influence the MIC values of benzalkonium chloride and chlorhexidine digluconate [19]. This observation was confirmed by our study showing that the MIC values were higher for chlorhexidine digluconate and benzalkonium chloride affected by QacE and QacEΔ1 efflux pumps in contrast to low MIC values of 2-phenoxy-ethanol, which is not affected by the efflux pump.

The disinfectant resistance of *A. baumannii* has been reported earlier, and it has been concluded that *qacE* and *qacEΔ1* genes in *A. baumannii* confer resistance to QACs [53]. The two genes, *qacE* and *qacEΔ1*, encode transmembrane proteins, and these are responsible for adaptation and tolerance to QACs in *A. baumannii*. Additionally, resistance to other types of biocides can be enhanced by them, enabling bacterial pathogens to survive and persist in a hospital environment [20]. It is important that both *qacE* and *qacEΔ1* genes are usually located on integrons and on plasmids that can be transferred by horizontal gene transfer between different Gram-negative bacteria (e.g., in *A. baumannii*) [53,54,55].

As far as we understand, this is the first report on the genes responsible for disinfectant resistance. More precisely, *qacE* and *qacEΔ1* were detected during the whole-genome analysis of the outbreak CRAB strain. The *qacE* and qacEΔ1 genes are not only responsible for the observed reduced sensitivity to disinfectants, but QAC disinfectants are able to induce the expression of efflux pumps at a tolerable disinfectant concentration, for which expression induction is concentration-dependent. Understanding this phenomenon is extremely important since cleaning and disinfection techniques are usually largely dependent on the cleaning individuals and processes, and the use of biocides at an inappropriate concentration can lead not only to the overgrowth of resistant strains but to a further increase in the strains’ resistance to disinfectants. However, the increased expression of the efflux pumps can induce resistance to various antibiotics through cross-resistance, representing an additional treatment failure and hospital outbreaks.

Appreciating this finding, it is very important to conclude that, in addition to antibiotic stewardship, the policy for disinfectant use should be implemented as part of infection-control activities. Responsible antibiotic policy and outbreak management must include a responsible disinfectant policy recognizing the spread of resistance.

Our study has inherent limitations. First, there was no implemented screening of healthcare workers for multi-resistant pathogens during the outbreak period. Second, no air sampling and analysis were carried out in the intensive care unit. Another limitation, if it can be called a limitation and not a strength, is that only a few vulnerable patients were affected by CRAB, so it was not possible to perform a statistical analysis of the risk factors. Furthermore, it would be worth examining the susceptibility and biofilm inhibitory effect of additional disinfectants, as in this study, only the effectiveness of the disinfectants used in the intensive care unit was investigated.

## 4. Materials and Methods

### 4.1. Study Design

In our retrospective cohort study, we described the epidemiological investigations and infection-control measures during a CRAB outbreak at the 175-bed Department of Neurosurgery and Neurointervention, Semmelweis University, Budapest, Hungary. The neurosurgical intensive care unit (ICU) comprises 18 beds with three5-bed rooms and three1-bed rooms. The patients housed here are with acute neurological disorders or after neurosurgical procedures. One of the 5-bed rooms is used for the 24 h observation of postoperative patients. Two hundred and ninety-one patients were treated together with postoperative patients in the ICU during the study period between 28 December 2023 and 22 February 2024.

Descriptive analysis summarized patient demographics, neurological diseases, surgical treatment, underlying diseases, infections, and outcomes.

### 4.2. Environmental Sampling

Environmental microbiological investigations were conducted in both the ICU and operating rooms in relation to the surgical procedures. Environmental samples were collected from frequently touched surfaces in the patient zone (e.g., respirators and monitors), shared areas (e.g., medication preparation area and storage), medical devices (e.g., anesthesia machine), and sinks (sink and sink drain) at the ICU and in the operating rooms as well. Between December 2023 and February 2024, a total of 174 environmental samples were collected from the ICU and the operating rooms. Sampling was repeated several times.

### 4.3. Bacterial Strain

During the outbreak, six *A. baumannii* strains were isolated from the patients. All *A. baumannii* isolates were identified by MALDI-TOF/MS (Bruker Daltonik GmbH, Bremen, Germany). Antibacterial susceptibility testing was performed on all strains, and the results were interpreted according to EUCAST guidelines (www.eucast.org), accessed 1 January 2024.

The MALDI-TOF/MS system was not used for epidemiological purposes in the routine clinical microbiology laboratory. Rather, single-nucleotide polymorphism (SNP) analysis and epidemiological data from The National Center for Public Health and Pharmacy, Budapest, Hungary, revealed a putative outbreak clone comprising six CRAB strains belonging to the globally disseminated international clone ST2. These strains had no SNP differences and had identical antimicrobial resistance and virulence genes.

### 4.4. Minimal Inhibitory Concentration and Minimal Bactericidal Concentration Determination

In order to assess the effectiveness of different disinfectants, a microdilution assay was carried out. Two hundred μL of bacterial broth was added into each well of a 96-well plate. These broth wells were then treated with varying concentrations (ranging from 128 mg/mL to 0.06 μg/mL) of benzalkonium chloride and chlorhexidine digluconate. The wells were subsequently incubated for 24 h at 37 °C. After 24 h, the minimal inhibitory concentrations were determined. MIC endpoint is the lowest concentration of disinfectants where no visible growth is seen in the wells. After the MIC determination aliquots, wells showing no visible bacterial growth were seeded on BHI agar plates and incubated for 24 h at 37 °C. The lowest concentration of an antimicrobial agent, when 99.9% of the bacterial population is killed, is termed the MBC endpoint.

### 4.5. Killing Curve Assay

We made two-fold serial dilutions of the active compounds of different disinfectants in Müller–Hinton Broth, and 200 µL was transferred to a 96-well microtiter plate in duplicate. As positive control of the culture medium, Müller–Hinton Broth was used. AMcFarland 0.5 suspension was prepared in MH Broth from overnight colonies of *A. baumannii* cultured on Columbia Blood Agar Medium, and the suspension was later diluted 1:20 in MH Broth for optimal bacterial density. As an inoculum, 20 µL of this diluted suspension was added to each well, except for negative controls. The plate was then incubated at 37 °C for 24 h, and the optical density at 595 nm was recorded every 15 min. However, some antibacterial agents cause the elevation of the turbidity spontaneously, as on optical background for distraction, we used the MH Broth containing the corresponding concentration of every antibacterial compound. Negative controls and background controls were adjusted with 20 µL of MH Broth without bacteria.

### 4.6. Investigation of Biofilm Production

To detect the biofilm formation, we followed the protocol reported by Hassan et al. [43]. Bacteria were incubated in EMEM medium (Lonza Bioscience, Budapest, Hungary) at 37 °C for 24 h. The cultures were then diluted 1:100 with fresh medium. Individual wells of sterile flat-bottom 96-well polystyrene tissue culture plates (Biologix Ltd., Hallbergmoos, Germany) were filled with 200 μL of the diluted cultures. Control *A. baumannii* BAA-1805 was similarly incubated, diluted, and added to the tissue culture plate. Negative control wells only contained inoculated sterile broth. The plates were incubated at 37 °C for 48 h. After incubation, contents of each well were removed by gentle tapping. The wells were washed with 0.2 mL of phosphate-buffered saline (pH 7.2) four times. This treatment removed free-floating bacteria. Biofilm formed by bacteria adherent to the wells was fixed by 2% sodium acetate and stained by crystal violet (0.1%). Excess stain was removed by using deionized water, and plates were kept for drying. The optical density (OD) of the stained adherent biofilm was read on a Multiskan FC Microplate Photometer (Thermo Scientific, Budapest, Hungary) at 595 nm. The experiment was performed in triplicate and repeated three times. The interpretation of biofilm production followed the criteria of Stepanovic et al. [56]. To calculate classification breakpoints, the following breakpoints were set during our analyses: OD control = 0.153, non-biofilm producer: OD ≤ 0.153, weak biofilm producer: 0.306 ≥ OD > 0.153, medium biofilm producer: 0.617 ≥ OD > 0.306, and strong biofilm producer: OD > 0.617.

### 4.7. Minimum Biofilm Inhibitory Concentration (MBIC) Determination

To detect the minimum biofilm inhibitory concentration, the method described above was used with the following modifications. Bacteria were incubated in EMEM medium (Lonza Bioscience, Budapest, Hungary) at 37 °C for 24 h. Cultures were then diluted 1:100 with fresh medium. Individual wells of sterile flat bottom 96 well polystyrene tissue culture plates (Biologix Ltd.) were filled with 200 μL of the diluted cultures. The broth wells were then treated with varying concentrations (ranging from 128 mg/mL to 0.06 μg/mL) of serially diluted benzalkonium chloride and chlorhexidine digluconate. The plates were subsequently incubated for 24 h at 37 °C. Control *A. baumannii* BAA-1805 was also incubated, diluted, and added to the tissue culture plate. Negative control wells contained inoculated sterile broth. The plates were incubated at 37 °C for 48 h. After incubation, contents of each well were removed by gentle tapping. The wells were washed with 0.2 mL of phosphate-buffered saline (pH 7.2) four times. This procedure removed free-floating bacteria. Biofilm formed by bacteria adherent to the wells was fixed with 2% sodium acetate and stained with crystal violet (0.1%). Excess stain was removed with deionized water, and plates were kept for drying. The optical density (OD) of the stained adherent biofilm was read on a Multiskan FC Microplate Photometer (Thermo Scientific, Budapest, Hungary) at 595 nm. The experiment was performed in triplicate and repeated three times. The interpretation of biofilm production was performed according to Stepanovic et al. [56]. The lowest concentration of benzalkonium chloride, chlorhexidine digluconate necessary to inhibit bacterial biofilm formation was identified as the minimal biofilm inhibitory concentration.

### 4.8. Whole-Genome Sequencing (WGS)

WGS analysis was performed on one *A. baumannii* strain (MACI-KO) by using the Illumina MiSeq system in Eurofins BIOMI Kft. (Gödöllő, Hungary). Briefly, genomic DNA was extracted with the NucleoSpin Microbial DNA Mini kit (Macherey-Nagel, Düren, Germany). The amount of isolated DNA was measured by Qubit fluorometer, and the quality of DNA was tested by microcapillary electrophoresis (Tape Station 4150, Agilent, Waldbronn, Germany). Libraries were prepared with the Illumina DNA Prep kit according to the manufacturer’s instructions. Sequencing was performed on an Illumina Miseq system using a MiSeq Reagent Kit v2, generating 250 bp paired-end reads. Genome assembly was performed with the SPAdes Genome assembler algorithm v. 3.15.3. [57]. Antibiotic-resistance genes were detected in the assembled genome using Bionumerics v. 8.1 software. The visualization was created with the Proksee server (https://proksee.ca/, accessed on 26 July 2024, London, UK) [58], and it is based on genome annotation data generated by RAST (https://rast.nmpdr.org/rast.cgi, accessed on 9 October 2020, Chicago, IL, USA) [59,60]. The assembled genome of *A. baumannii* strain was analyzed using the traditional 7-gene multilocus sequence typing (MLST).

### 4.9. Analysis of Disinfectant Resistance Genes by Quantitative PCR

The *A. baumannii* strain was co-cultivated with 0.0039% and 0.0078% concentrations of MBF at 0.0039% and 0.0078% concentrations of IP disinfecting agents in a 96-well microtiter plate at 37 °C. After overnight incubation, the total RNA of the bacteria was isolated with an innuPREP RNA Mini Kit 2.0 (Analytik Jena GmbH, Hardegsen, Germany) according to manufacturer’s instructions. RNA concentrations were determined using a NanoDrop 1000 spectrophotometer (Thermo Fisher Scientific, Budapest, Hungary). Ten to one hundred ng of RNA was used for RT-PCR assay performed using the PrimeScript RT reagent kit (Takara Bio, Saint-Germain-en-Laye, France) and amplified the resulting cDNA on a qTOWER 3G (Analytik Jena GmbH, Hardegsen, Germany) instrument in the presence of selected primers.

The primers for *qacE1* genes were 5′-GTATTGGCGACCGCTTTTCTCG-3′ forward and 5′-AGCCGACTGTAATAAAACCAATCCC-3′reverse. The primers for *qacEΔ1* were 5′-TGCTTATGCAGTCTGGTCGGG-3′ forward and 5′-ACCTACAAAGCCCCACGCATC-3′ reverse. The primers for *16S* were 5′-CATGCCGCGTGTGTGAAGAAG-3′ forward and 5′-AGCCGGTGCTTATTCTGCGAG-3′ reverse. Relative mRNA expression was calculated by means of the change in cycle threshold (ΔΔCt) and normalized to the geometric mean of housekeeping gene 16S. Basal mRNA levels of QacE1 and QacEΔ1 were compared with those of housekeeping gene 16S by calculating the difference between their Ct.

## 5. Conclusions

Here, we demonstrate that a complex infection control approach is important to effectively control the spread of nosocomial pathogens during an outbreak. We highlight the role of routinely used disinfectant agents during the cleaning procedures. Our work also highlights that considering disinfectant tolerance and resistance is important in the control of hospital epidemics. We recommend setting up phenotypic disinfectant susceptibility test standardization for outbreak strains causing hospital outbreaks. Whole-genome sequencing analysis of the outbreak strains is also useful. Or, for laboratories with limited resources, at least PCR-based rapid detection of *qacE1* and *qacEΔ1*, widespread mobile disinfectant resistance genes of Gram-negative HAI pathogens, would be desirable.

## Figures and Tables

**Table 1:** Characteristics of all the patients treated in the neurosurgical ICU during the study period.

Variables	All Patients (n = 291)
Age (years)	55.4 (38–85)
Gender, male	135 (46%)
**Neurosurgical disease**	
CNS tumor	175 (60%)
Subarachnoid hemorrhage	62 (21%)
Other hemorrhages	22 (46%)
Stroke	32 (7%)
**Surgical treatment**	
Neurosurgical operation	207 (71%)
Endovascular treatment	84 (29%)
**Underlying diseases**	
Hypertension	42 (14%)
Diabetes mellitus	49 (16%)
Others	64 (22%)
**Infection**	
Urinary tract infection	34 (11.6%)
Pneumonia	19 (7%)
Wound infection	10 (3.4%)
Bloodstream infection	7 (2%)
Meningitis	3 (1%)
**Death**	12 (4%)

CNS: central nervus system.

**Table 2:** Characteristics of CRAB-positive patients.

Patient	Age (Years)	Gender	Underlying Diseases	Surgical Procedure	Multiresistant Bacteria	CRAB-Positive Specimens	Infection/Colonization	Antibiotic Treatment	Duration of Hospital Stay (Days)	Outcome
P1	37	male	intracranial hemorrhage, hypertension	craniotomy	CRAB, MRSA	trachea	colonization	imipenem/cilastatin	29	discharged
P2	63	male	subarachnoid hemorrhage, hypertension	craniotomy	CRAB, VRE	urine, liquor, anorectal	infection: UTI, meningitis	colomycin, imipenem/cilastatin	58	discharged
P3	48	female	stroke, hypertension	intervention	CRAB	bloodculture	infection: BSI	colomycin	12	discharged
P4	67	female	AVM angiomatis, hypertension, diabetes mellitus, COPD	craniotomy	CRAB	trachea	colonization	imipenem/cilastatin, vancomycin, meropenem	6	discharged
P5	76	female	cerebral metastasis, hypertension, hypothyreosis, breast cancer	craniotomy	CRAB, ESBL-KP, VRE	wound, nasal, bloodculture	infection: SSI, BSI	colomycin, meropenem, vancomycin, linezolid, gentamycin	36	exit
P6	53	male	subarachnoid hemorrhage, hypertension, diabetes mellitus	craniotomy	CRAB, ESBL-KP, VRE	trachea, bloodculture, anorectal	infection: VAP, BSI	colomycin, ceftriaxone, cefatizidime, imipenem/cilastatin, meropenem, levofloxacin	38	discharged

CRAB: carbapenem-resistant *Acinetobacter baumannii*, MRSA: methicillin-resistant Staphylococcus aureus, VRE: vancomycin-resistant Enterococcus, ESBL-KP: extended-spectrum beta-lactamase producing K. pneumoniae. AVM: arteriovenous malformation, COPD: chronic obstructive pulmonary disease; UTI: urinary tract infection, BSI: bloodstream infection, VAP: ventilator-associated pneumonia.

**Table 3:** Summary of the infection-control strategy for CRAB outbreak.

	Before CRAB Outbreak	During CRAB Outbreak	After CRAB Outbreak
**Enforcement of hand hygiene**			
Regular education programs on hand hygiene	yearly	weekly, including circulating staff	every six months
Audit of hand hygiene compliance	every six month	daily	weekly
**Universal contact precautions**			
earing gloves, coat, apron, and shoe protector in the patient zone	in case of MDR-positive patients	in case of MDR-positive patients and their contacts	in case of MDR-positive patients and their contacts
Wearing of personal protective equipment by HCWs	yes	yes	yes
Access restrictions	yes	yes	yes
Review of invasive procedures	yes	yes	yes
**Cohort isolation**			
cohort isolation of colonized patient	no	yes	yes
Healthcare worker cohorting (HCW), patient:HCW ratio	3:1	2:1	3:1
**Surveillance cultures**			
Passive surveillance: antibiogram monitoring	yes	yes	yes
Active surveillance: screening cultures	no	initial (in 24 h) and in every 5 days	initial (in 24 h) and weekly
Environmental surveillance cultures	occasionally	once a week	every three months
**Patient decolonization**			
Throat decolonizitation with chlorhexidine digluconate	no	yes	yes
**Environmental cleaning and disinfection**			
Extensive management using checklists under the supervision of the unit manager	no	yes	yes
Patient zone	twice a day	three times a day, and necessery	three times a day, and necessery
Environment except the patient zone	twice a day	three times a day	three times a day
Cleaning personnel	two persons for the unit	three cohorting cleaning persons for the unit	three cohorting cleaning persons for the unit
Improved waste management	yes	yes	yes
Touchless environmental disinfection systems (e.g., vaporized hydrogen peroxide)	yes	yes	yes
**Developing a purposeful disinfectant policy**			
Disinfection stewardship	no	**Disinfection switch**	yes
**Antibiotic stewardship**	yes	yes	yes
**Genomic surveillance**			
Whole-genome sequencing	no	yes	no
**Monitoring and feedback**			
Discussions on the performance of the infection-control strategy	monthly	daily	weekly

**Table 4:** The minimum inhibitory concentration (MIC), the minimum bactericidal concentration (MBC), and the minimum biofilm inhibitory concentration (MBIC) of the outbreak CRAB strain (µg/mL).

Disinfectants	*Acinetobacter baumannii*				*Acinetobacter baumannii*			
	Outbreak CRAB				BAA-1805			
	MIC	MBC	MBC/MIC	MBIC	MIC	MBC	MBC/MIC	MBIC
Benzalkonium chloride	32	32	1	0.5	16	32	2	<0.06
2-phenoxyethanol	0.5	0.25	2	0.0078	0.25	0.25	1	0.0078
Chlorhexidine digluconate	16	32	2	0.25	32	64	2	8

**Table 5:** Characteristics of genome sequence of *A. baumannii* (JBEVZX010000001).

**Multilocus sequence typing (Pasteur schema)**	ST2
**Beta-lactamases**	*bla*_OXA-83_ (OXA-51-like)
	*bla*_ADC-30_
**Fluoroquinolone resistance determinants**	*gyrA*: Ser81Leu *parC*: Ser84Leu
**Aminoglycoside resistance genes**	*ant(3″)-II* (aminoglycoside nucleotidyltransferase) *aph(6)-Id* (aminoglycoside O-phosphotransferase) *aph(3″)-Ib* (aminoglycoside O-phosphotransferase) *aac(6’)-Ia* (aminoglycoside 6′-N-acetyltransferase)
**Efflux pumps**	*adeABC*; *adeFGH*; *adeIJK*; *adeRS*; *adeL*; *adeN*
**Disinfectant, antiseptic resistance determinants**	*qacE*; *qacEΔ1*
**Biofilm formation**	*pgaABCD*; *csuA/B*, *csuABCDE*; *bfmR*; *bfmS*; *ompA*

## Data Availability

The genome sequence of the *A. baumannii* strain (MACI-KO) is deposited in the NCBI database at the following bioproject accession number: PRJNA1130157.
